# Shared Decision-Making in the Management of Deep Partial-Thickness Burns: Development and Implementation of a Multicomponent Decision Aid

**DOI:** 10.2196/86789

**Published:** 2026-09-04

**Authors:** Roos F C Salemans, Robin A F Verwilligen, Sven J G Geelen, Margriet E van Baar, Regina The, Irma Visser, Karlijn F Joosten, Ymke Lucas, Annebeth Meij-de Vries, Kiran Baran, Sonja M H J Scholten-Jaegers, Cornelis H van der Vlies, Denise van Uden, National Burn Care, Education & Research group the Netherlands

**Affiliations:** 1 Trauma Research Unit Department of Surgery, Erasmus MC, University Medical Centre Rotterdam, PO Box 2040, Dr. Molewaterplein 40, Rotterdam, 3000 CA, The Netherlands, 31 102913349; 2 Alliance of Dutch Burn Care (ADBC), Burn Centre, Maasstad Hospital, Rotterdam, The Netherlands; 3 Alliance of Dutch Burn Care (ADBC), Burn Centre, Red Cross Hospital, Beverwijk, The Netherlands; 4 Amsterdam UMC location VUmc, Department of Plastic, Reconstructive and Hand Surgery, Amsterdam, The Netherlands; 5 Amsterdam Movement Sciences (AMS), Tissue Function and Regeneration, Amsterdam UMC, Amsterdam, The Netherlands; 6 Alliance of Dutch Burn Care (ADBC), Burn Centre, Martini Hospital, Groningen, The Netherlands; 7 Research group Healthy Ageing, Allied Health Care and Nursing, Hanze University of Applied Sciences, Groningen, The Netherlands; 8 ZorgKeuzeLab, Delft, The Netherlands; 9 Dutch Association of Burn Survivors, Beverwijk, The Netherlands; 10 Alliance of Dutch Burn Care (ADBC), Dutch Burns Foundation, Beverwijk, The Netherlands; 11 Department of Surgery, Red Cross Hospital, Beverwijk, The Netherlands; 12 Amsterdam UMC location University of Amsterdam, Pediatric Surgical Centre, Emma Children’s Hospital, Amsterdam, The Netherlands; 13 Departments of Trauma and Burn Surgery, Maasstad Hospital, Rotterdam, The Netherlands; 14 see acknowledgements

**Keywords:** burns, burn surgery, decision aid, shared decision-making, value-based health care

## Abstract

**Background:**

In burn care, one of the most debated topics is the optimal treatment of patients with deep partial-thickness burns. With these patients, the decision must be made to perform early surgery or to wait and potentially limit, or even avoid, surgery. Both options are available in Dutch burn care, and the best treatment option is decided on clinical outcomes as well as patients’ preferences. This complexity highlights the need for shared decision-making (SDM) and a decision aid (DA) to facilitate this process.

**Objective:**

This study aimed to support patients and health care professionals (HPs) in the process of SDM regarding the treatment decision for deep partial-thickness burns by developing and implementing a DA.

**Methods:**

This multimethod design was conducted in a Dutch burn care setting between September 2023 and October 2025 and included 3 phases. Phase 1 (needs assessment) included semistructured interviews with patients, analyzed using a qualitative descriptive approach with deductive and inductive coding, and an online survey of HPs with a descriptive analysis of closed-ended questions and inductive content analysis of open-ended questions by 2 researchers. Phase 2 (development) involved 5 co-design sessions with patients, HPs, and researchers. The think-aloud method was used for usability testing with patients and HPs. Additionally, HPs participated in acceptability testing using interviews guided by the Consolidated Framework for Implementation Research, and analyzed using deductive thematic analysis. Phase 3 (implementation) consisted of a 6-month pilot period, including patient interviews, 3 focus groups with HPs, usage data, and the Normalization Measure Development questionnaire to assess the level of normalization.

**Results:**

Eight patient interviews revealed 2 distinct information needs: patients seeking detailed treatment information and those preferring to defer decisions to clinicians. The HPs survey (response rate 36%) showed that burn physicians typically make the final treatment decisions, although SDM was considered preferable, and 91.2% (31/34) supported the use of a DA. Usability testing with 4 patients and 7 HPs showed overall satisfaction, with minor revisions suggested, such as clarifying text and illustrations. Key implementation facilitators included professional engagement and local support, while infrastructure was the main barrier. The final DA comprised a paper handout sheet, an interactive website, and a summary sheet capturing patient preferences. During the pilot, the DA was distributed 42 times and used by 28 patients (67% participation rate). Both patients and HPs reported positive experiences, and the tool was considered feasible to integrate into routine care.

**Conclusions:**

A DA for the treatment of deep partial-thickness burns was successfully developed and implemented using a comprehensive, user-centered approach. It supports SDM and patient-centered care by providing tailored information and helping patients participate more actively in treatment decisions.

## Introduction

Burn injuries represent a considerable public health concern worldwide as they cause a substantial burden of disease. An important characteristic of a burn injury is its healing potential, as it guides optimal treatment [1]. To accurately determine the healing potential, one can use laser Doppler imaging (LDI), which can derive the healing potential from a wound by measuring dermal perfusion [2,3]. Using LDI in combination with clinical assessment gives a good indication of whether surgery might be needed; however, this is not as evident for all burns. In burn care, one of the most debated issues is the optimal treatment of deep partial-thickness burns [4]. The treatment of these burns frequently involves debridement of the damaged tissue, followed by autologous skin grafting [5]. On the other hand, conservative treatment can also be applied, with the potential to delay or avoid the need for surgical intervention or limit the extent of excision [6]. Clinical assessment after 2 to 3 weeks will indicate whether surgical intervention is still preferred. In short, when surgical intervention is applied, there is no consensus on the ideal timing for this approach. Currently, Dutch specialized burn care leans toward a more conservative approach with a median timing of 14 days post burn to surgery [7]. The most appropriate treatment option is decided on clinical outcomes as well as patients’ preferences. Early surgical intervention, aimed at rapid wound closure, has been associated with reduced risk of infection, shorter healing times, and faster overall recovery [8,9]. On the other hand, it can also result in excessive excision of more superficial burn areas and increased blood loss, creating a donor site or an increased risk of additional surgeries. Delaying surgery can reduce the surgical surface or even eliminate the necessity for a surgical intervention [10]. From a patient perspective, the ideal timing of the treatment may be influenced by factors such as hospitalization time, the burden of wound care, or the anxiety related to surgery. This complexity highlights the need for shared decision-making (SDM) between clinicians and patients with deep partial-thickness burns regarding the decision for early or “late or no” surgery.

The perspectives of clinicians as well as patient values and preferences play an important role in treatment decision-making. However, SDM can especially enhance patient involvement and autonomy [11]. It adds to the value-based health care principle by better aligning with patients’ needs and therefore potentially improves specific outcomes that matter most to the patient. Nevertheless, the implementation of SDM presents certain barriers. Waddell et al [12] conducted a systematic review of the barriers of SDM in hospital settings. Barriers identified included lack of SDM training, clinician concerns about patient outcomes, perception of acuity limiting SDM, and lack of time or space. Barriers focusing on the acuity of care and the lack of time or space to apply SDM might be particularly relevant for burn care, as this is considered to be acute complex care and highly specialized, involving multiple phases and multidisciplinary fields [13]. Waddell et al [12] also identified facilitators of SDM, which included provision of tailored risk and benefit information, clear interprofessional communication, clinician awareness of biases, and understanding patient and family needs.

When implemented correctly, SDM has the potential to strengthen the relationship between patients and health care professionals (HPs) by facilitating more informed decision-making, ultimately contributing to improved patient outcomes [14]. A widely adopted approach to SDM is the “three-talk model” by Elwyn et al [15], consisting of (1) a team talk; (2) an option talk; and (3) a decision talk. The integration of evidence-based information together with the patients’ values and priorities makes SDM the preferred approach among patients, as it enhances patient satisfaction, therapy compliance, and commitment, while also increasing patient knowledge and involvement [14-18]. Conversely, it reduces doubt, regret, costs, and the number of operations.

Decision aids (DAs) are effective tools to provide evidence-based information, clarify patients’ needs and preferences, and promote the use of the three-talk model in clinical practice [15,16]. This enables SDM and an active role for patients in making health care decisions [19]. DAs have been shown to increase knowledge, improve risk perception, and align treatment choices with patient priorities [16]. For HPs, enhanced communication is supported by the DA through structured guidance by a stepwise framework and consistent messaging, thereby further facilitating SDM for both parties [16]. Despite the aforementioned benefits, DAs are not used to their full potential in burn care. Several decision-support tools have been developed for specific populations and burn types [20-23], but to the best of our knowledge, no DA has been specifically designed for the treatment of patients with deep partial-thickness burns.

This study aimed to provide support to patients with deep partial-thickness burns and HPs in making the decision between early surgery or “late or no” surgery by developing and implementing a novel DA. This DA aims to support the SDM process and contribute to patient-centered care in the treatment of acute burns.

## Methods

### Ethical Considerations

The MEC-U (Medical Research Ethics Committees United; numbers W23.192 and W24.066) and review boards of the participating hospitals (Red Cross Hospital Beverwijk, Martini Hospital Groningen, and Maasstad Hospital Rotterdam) approved this study. This study was conducted in accordance with the principles of the Declaration of Helsinki. Informed consent was obtained from all patients and HPs before participation. Oral consent was provided and recorded for the patient needs assessment interviews, the usability testing with patients and HPs, acceptability testing with HPs, the evaluation interviews with patients, and the evaluation focus groups with HPs. Digital informed consent was provided for the needs assessment survey and the Normalization Measure Development (NOMAD) questionnaire.

All study data were coded and stored digitally on a secured network drive accessible only to the dedicated research team. A key file linking codes to participant identities was stored separately and protected with a password to ensure confidentiality. Patients participating in the co-design session were compensated €100 (with a currency exchange rate in January 2024 of €1=US $1.091) per session for their contribution, and patients completing the usability test were compensated with a €25 gift card.

### Study Design

The DA was developed and implemented using a multimethods design. The development and implementation comprised three phases: (1) a needs assessment, (2) development of the DA by co-design sessions, and usability and acceptability testing, and (3) implementation by pilot testing and evaluation. All phases were facilitated and supported by ZorgKeuzeLab B.V. in Delft, the Netherlands (development and implementation partner who has extensive experience in DAs).

The organization structure consisted of a dedicated project team responsible for the coordination and supervision of the development and implementation process; a steering group involved in the co-design sessions that was responsible for the scope, content, and design of the DA; an HP panel and patient panel involved in the needs assessment, usability, and acceptability testing of the DA; and an implementation team at each burn center.

### Recruitment and Sampling

Mentally competent adults with sufficient Dutch language skills who had sustained deep partial-thickness burns and were treated at the Maasstad Hospital Rotterdam, Martini Hospital Groningen, or Red Cross Hospital Beverwijk burn center were eligible to participate in this study. For the usability test of phase 2, patients had to be treated within the past 4 years in one of the burn centers. For the co-design sessions of phase 2, only members of the patient advisory committee were recruited. Eligibility for the patient evaluation interviews in phase 3 required that patients had used the DA during the 6-month pilot period (November 5, 2024, to May 1, 2025), allowing opportunistic selection of those who provided relevant feedback. The number of patients, HPs, and researchers participating in each study component is summarized in Table 1. Throughout this study, purposive sampling was used, with a different strategy used for each study component, depending on whether the aim was to capture variation or focus on similarities [24].

For the phase 1 needs assessment interviews with patients, a purposive sampling with a maximum variation strategy was applied to explore potential differences in information needs, taking patient characteristics such as background, level of education, and age into account. This provided a mixed group of patients with different characteristics. Patients for the co-design sessions were selected using homogeneous sampling, as only patients of the patient advisory committee were eligible. This group was intentionally chosen because they share similar experiences with patient participation, familiarity with burn care, and the ability to reflect on information needs from a patient perspective. Patients for the usability testing were selected using a purposive criterion sampling strategy, including only patients who sustained deep partial-thickness burns within the past 4 years. For the evaluation interviews, sampling followed a purposive opportunistic sampling strategy; an HP and researcher identified patients who had provided relevant feedback during the use of the DA and were therefore considered most suitable to inform the evaluation.

Patient selection was done by both the treating HP and the dedicated researcher. After identification, the HP approached the patient and, upon agreement, the researcher informed the participant about the aim and procedures of this study and scheduled the interview. No personal characteristics, assumptions, or personal motivations were shared.

**Table 1.** Number of participating patients and health care professionals in the various study components.

Study component	Patients participating, n	Health care professionals participating, n	Researchers participating, n
Phase 1			
Needs assessment interview	8	N/A^a^	N/A
Needs assessment survey	N/A	40	N/A
Phase 2			
Co-design session	2	10	4
Usability testing	4	7	N/A
Acceptability testing	N/A	7	N/A
Phase 3			
Evaluation interview	4	N/A	N/A
Evaluation focus group	N/A	7	N/A
Normalization Measure Development Questionnaire	N/A	13	N/A

a N/A: not applicable.

Eligible HPs for the needs assessment survey had to be working at 1 of the 3 burn centers and were identified through complete departmental mailing lists provided by this project’s coordinators of each burn center (DvU, RAFV, and SJGG). A complete sampling approach was used to capture perspectives across different professionals, years of experience, and burn centers. HPs were invited by email. Eligible HPs in the co-design sessions, usability, acceptability, and evaluation with HPs had to be working at 1 of the 3 burn centers and be involved in care and decision-making for deep partial-thickness burns. A criterion-based strategy was used to target HPs working in Dutch burn care who are involved in decision-making and the implementation of the DA. These HPs were approached directly by the researcher.

No formal sample size calculation was performed as no reliable prior data were available to estimate the expected effect size. The time period for recruitment was September 2023 until October 2025.

### Data Collection and Analyses

In all phases, Amberscript was used to transcribe the audio recordings of the interviews. Necessary corrections were made by researchers RFCS and DvU for the needs assessment interviews and by researcher DvU for the evaluation interviews. For the usability and acceptability testing, Microsoft Teams (version 2023) was used to transcribe the video recordings, and field notes were taken during testing. Online surveys were completed using Castor Electronic Data Capture (version 2024.4.1.1). MaxQDA (VERBI software GmbH, version 24.6) was used for qualitative analyses. All quantitative data were analyzed with IBM SPSS (version 29).

### Phase 1: Needs Assessment Among Patients With Burns and HPs

#### Patient Interviews

Two postdoctoral female researchers (RAFV and DvU) conducted in-depth semistructured interviews with patients. No one other than the researcher and participant was present during data collection, and field notes were taken during the interviews. The interviews are reported in accordance with the COREQ (Consolidated Criteria for Reporting Qualitative Research) checklist. The COREQ checklist and interview guide are included in Checklist 1. Patients were questioned about their views on the current information provision and their need for information to make a well-informed decision regarding the treatment of their partial-thickness burn. The interview guide was created in collaboration with the experts of ZorgKeuzeLab and included questions regarding patient characteristics and medical background, experiences on the care trajectory and information delivery, needs in care trajectory and information delivery, experiences with SDM and aspects to consider, and involvement and needs of relatives. The interviews were conducted either by telephone or in person. All interviews were audio-recorded and transcribed verbatim. Data were analyzed using a qualitative descriptive approach with a combined deductive and inductive coding strategy. To ensure data familiarization, both researchers repeatedly read the transcripts. First, 2 interviews were coded in parallel to compare emerging codes, memos, and interpretations. Based on this, an initial coding approach was agreed upon. The 2 researchers (RFCS and DvU) then independently coded the remaining interviews, with each transcript coded by 1 researcher. A predefined definition of a “need,” adapted from Endacott, was used: “an undesirable state of affairs; a deficit; a necessity; its presence confers responsibility to make good the deficit; and evaluation notion (or value judgment) someone has to define them as needs” [25]. All segments that met this definition were coded as needs. Need-related codes were then discussed in consensus meetings when required and inductively grouped and summarized to identify and refine the overarching information needs.

#### HPs Survey

In addition, HPs took part in an online survey to determine their views on and experiences with current information provision, treatment choices, and SDM. The survey comprised both closed and open-ended questions on experiences regarding information delivery, treatment options, SDM, and the DA, and was developed in collaboration with the experts of ZorgKeuzeLab (Multimedia Appendix 1). Descriptive statistical analysis was used for closed-ended questions, with categorical data reported as numbers and percentages. Open-ended questions were analyzed using a qualitative descriptive approach with inductive category development. Two researchers (RFCS and DvU) independently reviewed and coded all open-ended responses to generate initial categories. These independently developed category sets were then compared and discussed until consensus was reached on the final categories, which were subsequently used to classify all open-ended responses. Finally, categories were ranked according to the frequency with which they were cited (Multimedia Appendix 2).

### Phase 2: Development of the DA

#### Co-Design Sessions

The steering group, consisting of patient representatives (n=2), HPs (n=10), and researchers (n=4), decided on the DA design and content in 5 co-design sessions. Field notes were taken during the co-design sessions and analyzed through a structured synthesis of session output to inform iterative design. During the first co-design session, results of the needs assessment were discussed with the steering group, and the objective, scope, and target patient group of the DA were defined. In session 2, the general outline of the DA, its integration into the clinic, and the content of the summary sheet were determined. The summary sheet, which is shown at the end of the DA, summarizes the patient’s values and preferences that were filled in while using the DA. This supports decision-making during consultation with the HP. In session 3, the DA structure was finalized, and the content was proofread. Subsequently, in session 4, the DA content was concluded, and the handout, which is used to indicate the patient’s burn diagnosis and treatment choices, was determined. Additionally, the sheet contained the name of the treating HP and a personal login for the interactive website. Finally, the results of the usability and acceptability testing were discussed, and final adjustments were made in session 5. The text of the DA was proofread and edited by a medical text writer to common Dutch (B1 level), and illustrations were specifically designed by an illustrator. The content followed the International Patient Decision Aid Standards criteria [19,26].

#### Usability Testing

In the usability test, 4 patients who had sustained deep partial-thickness burns and had been treated at one of the burn centers within the past 4 years, and 7 HPs (nurses, physicians, and surgeons) involved in treatment decisions for deep partial-thickness burns, tested the concept version of the DA through think-aloud sessions [27,28]. Patients were instructed to use the DA as though they were preparing for an upcoming consultation and to verbalize their thoughts while doing so. This enabled the researchers (RAFV and DvU) to follow their thought processes [28]. It was determined whether patients understood the content, whether the DA was easy to use, and whether they perceived added value. HPs reviewed the DA in advance and then walked through the content during the think-aloud session, providing detailed comments on clarity, completeness, and feasibility of integrating the DA into clinical workflow. The think-aloud sessions with patients and HPs were conducted by 2 researchers (RAFV and DvU), and in some cases, researcher KFJ was present to assist with field notes. Sessions were audio-recorded on Microsoft Teams (version 2023) and supplemented with field notes, allowing researchers to observe both verbal and nonverbal responses. A descriptive qualitative analysis focusing on usability issues was conducted by RAFV and DvU. Transcripts and field notes were reviewed to identify usability issues and contextual observations, document required adjustments to the DA, and select illustrative quotes. The main discussion points and necessary changes were identified and discussed with the steering group in the final co-design session.

#### Acceptability Testing

For the acceptability test with HPs, an interview on facilitators and barriers to using the DA was conducted, guided by the Consolidated Framework for Implementation Research (CFIR) [29]. CFIR-based questions were analyzed using a deductive thematic approach guided by CFIR: interview segments were coded against predefined CFIR domains and constructs by a single researcher (DvU). As CFIR provides a structured set of constructs, this deductive coding primarily involved assigning data to existing categories, with limited interpretive flexibility; therefore, this part of the analysis was conducted by 1 researcher (DvU). To identify facilitators and barriers perceived by HPs, predefined themes were determined by the CFIR framework, including innovation relative advantages, local attitudes, local conditions, infrastructure, culture, access to knowledge and information, and engagement. All responses were coded into the predefined categories, after which the information within the coded segments was summarized to reflect what HPs considered key facilitators and barriers influencing the implementation of the DA.

### Phase 3: Implementation of the DA

Subsequently, the DA was put into clinical practice using a multifaceted and tailored implementation strategy guided by the expertise of ZorgKeuzeLab and the CFIR-identified facilitators and barriers of phase 2. As personal engagement and local conditions were key facilitators, the implementation team was actively involved in shaping the implementation strategy, defining implementation goals, allocating tasks and responsibilities, and addressing logistics, which had been identified as a major barrier. The implementation began with an initiation meeting at each burn center 1 month before the start of the pilot. This meeting was attended by the dedicated implementation team, including HPs involved in handing out the DA. The meeting was led by 2 researchers (RAFV and DvU), with researcher KFJ to assist with field notes. Sessions were audio-recorded on Microsoft Teams (version 2023) and supplemented with field notes, allowing researchers to observe both verbal and nonverbal responses. Its purpose was to set the implementation goals, determine the patient journey for the DA, and divide tasks needed for successful implementation. The team collaboratively mapped the patient journey and determined how to integrate the DA into existing workflows. In addition, HPs received instructions on the practical use of the DA, including task assignments. Points of attention and implementation goals were formulated, and estimations for several indicators were made, such as the expected annual number of eligible patients. To raise awareness and encourage engagement among the entire burn care team, a kick-off meeting was held to introduce all HPs to the DA and its intended use in routine care, officially launching the pilot period. Broad involvement and knowledge among all HPs was considered essential, as all team members needed to be able to support patients who might have difficulty accessing or using the DA. Executive boards were involved to secure institutional support and strengthen commitment throughout the organization. To determine the implementation of the DA, it was pilot-tested for 6 months, starting on November 5, 2024, and ending on May 1, 2025. Throughout the pilot period, HPs received monthly reports that included the predefined implementation indicators, and interim evaluation meetings were held to allow the team to refine logistical processes and make adjustments as needed. All HPs were trained on SDM before the start of the implementation.

After the pilot period, evaluation with patients and HPs followed. A structured interview guide was used to evaluate patients. Interviews were conducted by telephone, audio-recorded, and transcribed verbatim. No one other than the researcher (DvU) and the participant was present during data collection, and field notes were taken. Data were analyzed using a descriptive thematic analysis of patients’ experiences of using the DA. The transcripts were reviewed by a single researcher (DvU), who coded segments describing positive and negative experiences and grouped these codes into descriptive themes. Illustrative quotes reflecting patients’ experiences with the DA were identified within each theme to exemplify the experiences. As this component aimed primarily to provide a descriptive summary of user experiences and illustrative quotations, a single-researcher analysis (DvU) was considered appropriate. Three focus groups were conducted with HPs to evaluate their experiences using the DA. Sessions were facilitated by 2 researchers (RAFV and DvU), recorded via Microsoft Teams, and supplemented with field notes. Subsequently, a descriptive thematic analysis was conducted by RFCS and DvU, in which codes were grouped into themes describing experiences with the DA. Both researchers independently reviewed the transcripts and coded statements reflecting HPs’ experiences with the DA. Codes were then discussed in consensus meetings to compare interpretations and resolve any discrepancies, after which they were grouped into descriptive themes summarizing HPs’ experiences. Additionally, during the pilot period, ZorgKeuzeLab collected usage data of the DA, including the number of patients who received and used it, the participation rate, and the time spent using it. The degree of normalization among HPs was assessed once at the end of the pilot using the NOMAD questionnaire [30]. The NOMAD questionnaire is a validated instrument based on Normalization Process Theory that measures the extent to which a complex intervention is embedded in routine practice from the perspective of HPs. The results were analyzed using descriptive statistical analysis, with the results presented as numbers and percentages.

## Results

### Phase 1: Needs Assessment Among Patients With Burns and HPs

#### Patients’ Informational Needs to Make a Well-Informed Decision

Eight former patients with deep partial-thickness burns, recruited from all 3 Dutch burn centers, participated in the needs assessment interviews. The mean age of the patients was 57.6 years (n=7, SD 15.0, range 29-75), and the majority of the patients were male in terms of gender (6/8, 75%). Most patients sustained flash or flame burns (7/8, 87.5%), primarily at home. More than half of the patients were first taken to a local hospital by ambulance (5/8, 62.5%), after which they were transferred to a burn center, while 37.5% (3/8) of patients were transferred to a burn center directly. The length of hospital stay ranged from 3 days to 4 weeks, and most patients underwent at least one surgical procedure (6/8, 75%).

The informational needs of patients to make a well-informed decision regarding the treatment of their burns varied. Patients could be divided into 2 groups, namely patients who were in need of information about their treatment and recovery and patients who did not seek this information.

Regarding patients who had a need for information, 2 main groups of patients could be identified (Table 2). First, patients who wish to receive information during the acute phase. Second, those who wish to receive information at a later stage. The reason for wanting to receive the information later was that, as time passed, patients had had more time to adjust to the situation and therefore felt able to ask questions.

Patients wanting to receive information in the acute phase mostly wanted to know what to expect in the future or needed more general information on treatment options and procedures, as was illustrated by the following quotes: “The information that you are looking for is mainly about what can I expect afterwards” and “I wanted to know what the advantages and disadvantages were.”

Patients who did not require information did so for different reasons. Some of these patients who displayed a more fatalistic or indifferent perspective believed that everything was beyond their control, and simply let life happen to them. A patient illustrated:

*That is who I am: just let everything happen to you, you know. And just wait what the doctors will plan and how or what they will do*.

**Table 2.** Summary of findings from the needs assessment among patients with burns.

Need for information	Phase of care	Description
Patients in need of information	Acute phase	Need to know what can be expected of wound recovery and scar formation in the future Explanation of the different treatment options and the general advantages and disadvantages of the treatment options
Patients in need of information	Later phase	In a later phase, patients are better able to focus on reading about burn treatment options, procedures, and recovery
Patients not in need of information	Acute phase	Patients in the acute phase feel the need to focus on their recovery and might not have the mental space to read about burn treatment options and their recovery
Patients not in need of information	Acute and later phase	Some patients had a more fatalistic or indifferent perspective Information provision is seen as sufficient

#### HP Views and Experiences on SDM

The survey was sent to 111 HPs, of whom 40 HPs filled out at least the general “about you” questions of the survey, which included questions regarding their position, years of experience, and affiliated hospital (response rate 36%). Most of the respondents had over 5 years of experience in burn care (27/40, 67.5%). Most of the HPs were nurses (19/40, 47.5%) or burn physicians (6/40, 15%). Burn physicians, trauma surgeons, and plastic surgeons were mostly involved in the treatment choice for early or “late or no” surgery for patients with deep partial-thickness burns (Table 3). The HP decides whether to discuss treatment options for deep partial-thickness burns with the patient, based on the severity of the burn (depth and the percentage total body surface area burned) and the patient’s overall health status, work or hobbies, preferences, and whether they are in pain. More than half of the HPs indicated that current information provision for HPs on these specific treatment options is insufficient. The majority of respondents indicated that the HP is the main source of information. Only a small number of respondents reported information sources such as literature, information leaflets, or digital information. Furthermore, when asked whether patients and their families are adequately informed on the treatment decision of early or “late or no” surgery, answers were divided: agreed or strongly agreed (17/36, 47.2%), disagreed or strongly disagreed (13/36, 36.1%), and neutral (6/36 16.7%). The burn physician usually makes the final treatment decision. However, the majority of HPs responded that ideally the final decision should be made jointly by the physician and the patient. When asked whether they would find a DA desirable, 91.2% (31/34) said they would. As mentioned in the open-ended answers, the major advantages of such a DA were increased autonomy for patients, better-informed patients, assisting patients in decision-making, and helping to tailor treatment. The main disadvantages mentioned were that the DA might not be suitable for all patients, and that it could cause logistical inconveniences. It could also place too much responsibility on patients, whose decision might not align with those of HPs. Patients may also receive too much or unclear information, and have insufficient time to go through the DA. The survey concluded with a question about which topics a DA should address regarding early or “late or no” surgery. HPs mostly mentioned that it was important to cover the advantages and disadvantages of the treatment options, followed by scar quality and the expected recovery time for the burn wound and donor site. They also mentioned the expected risks or complications, as well as the number of surgeries required for each treatment option.

**Table 3.** Needs assessment survey among health care professionals.

	Value
Health care professionals involved in the choice between early or “late or no” surgery^a^ (n=39), n (%)	
Burn physician	38 (97.4)
Trauma surgeon	24 (61.5)
Plastic surgeon	18 (46.2)
Nurse specialist	13 (33.3)
Resident	11 (28.2)
Nurse	7 (17.9)
Medical manager	2 (5.1)
Team leader	1 (2.6)
Other	2 (5.1)
Satisfaction with the current provision of information for the health care professional about the option early or “late or no” surgery (n=39), n (%)	
Not at all satisfied	5 (12.8)
Not satisfied	18 (46.2)
Not dissatisfied nor satisfied	14 (35.9)
Satisfied	2 (5.1)
Very satisfied	0 (0)
Decision on early or “late or no” surgery is usually made by^a^ (n=36), n (%)	
Burn physician	34 (94.4)
Trauma surgeon	13 (36.1)
Plastic surgeon	11 (30.6)
Nurse specialist	1 (2.8)
Medical manager	1 (2.8)
Resident	0 (0)
Nurse	0 (0)
Team leader	0 (0)
Other (the patient or their family)	10 (27.8)
Final decision for early or “late or no” surgery should ideally be made by (n=35), n (%)	
The physician and the patient and family together	24 (68.6)
The patient and family after hearing and considering the physician’s opinion	9 (25.7)
The physician after hearing and considering the opinion of the patient and family	2 (5.7)
The patient and family	0 (0)
The physician	0 (0)

a Multiple answers possible

### Phase 2: Development of the DA

#### Overview

**Figure 1.**
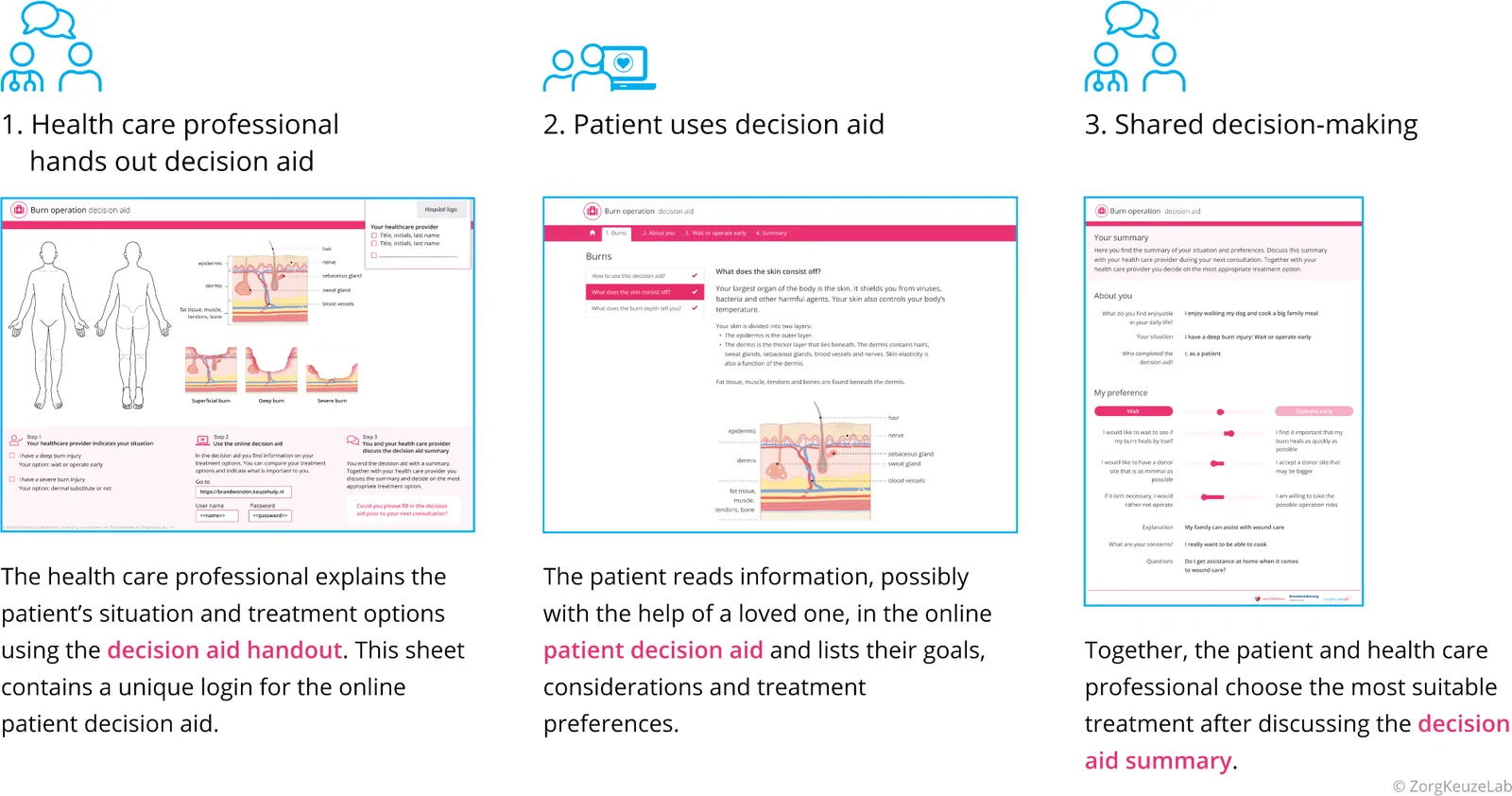
The final decision aid consists of three components: a paper handout sheet, an interactive website, and an online summary sheet.

During the co-design sessions, findings from the phase 1 needs assessment with patients and HPs were used to guide the content and outline of the DA. To address the varying informational needs of patients, an expand and collapse function was added, allowing patients to access additional information when desired. Patients indicated a need for general information on treatment options, procedures, and expected outcomes for each treatment option, which was therefore integrated into the DA. Likewise, topics highlighted by HPs in the survey were incorporated. HPs emphasized the importance of clearly presenting the advantages and disadvantages of each treatment option, as well as information on scar quality, recovery time, potential risks or complications, and the expected number of surgeries per treatment option. The 5 co-design sessions resulted in the final product, comprising a DA handout, an interactive website, and a DA summary sheet (Figure 1).

#### Usability Testing

Both patients and HPs were very satisfied with the proposed DA and indicated that the information given was clear, complete, and easy to understand. The majority of the patients and HPs felt the length of the interactive website was acceptable, and both patients and HPs mentioned that the current DA met their needs. Patients indicated that the information in the DA reflected their own disease experiences. One of the patients mentioned: “It looks good and very neat, very clear as well. I could relate to it.” Another patient mentioned: “It is perfect and user friendly*.*” The proposed changes included adjustments of text and illustrations, and clarifying information.

#### Acceptability Testing

##### Overview

The acceptability test with HPs involved a brief interview about their impressions and the facilitators and barriers of the DA, using the CFIR framework. Several facilitators and barriers were considered, and the most relevant ones were identified, as detailed below. In short, engagement and local conditions could facilitate implementation, whereas poor infrastructure was considered the most important barrier. Culture, referring to the norms and values of an organization, was considered a possible facilitator as well as a possible barrier.

##### Engagement

Personal engagement was considered a strong facilitator, with HPs indicating their willingness to implement and use the DA. Patient engagement, on the other hand, was seen as a possible barrier due to low health literacy.

##### Local Conditions

Local conditions, such as willingness and putting effort into using the DA, were seen as a strong facilitator. HPs indicated that they are generally able to hand out the DA, although this is somewhat dependent on their workload. Even so, they reported doing their utmost to find time to facilitate the patients in using the DA.

##### Infrastructure

Logistics and digital infrastructure were seen as the main barriers. Fitting the DA process into the current clinical and outpatient logistics was considered extremely challenging. Providing enough time for a patient to use the DA and discussing their preferences afterward, but also being able to facilitate the treatment options due to limited surgical capacity, was seen as challenging. Additionally, it was questioned whether patients would have the digital skills and support to log in to the website and use it properly.

##### Culture

While 1 HP mentioned a positive attitude toward the use of the DA among colleagues, another noted an initial stubborn attitude toward new initiatives. However, according to the HPs, this does not mean that the DA is deemed an unsuccessful implementation. It is therefore crucial to ensure that all HPs are aware of the innovation and are involved, so that it becomes part of their routine.

### Phase 3: Implementation of the DA

#### Overview

The facilitators and barriers identified in the CFIR-based interviews informed the multifaceted strategy used during the 6-month pilot period. This strategy included educational meetings, involvement of executive boards, efforts to promote adaptability, and reminders for clinicians throughout the process. After the pilot period, the implementation was evaluated using DA usage data, patient interviews, focus groups with HPs, and the NOMAD questionnaire.

#### DA Usage Data

During the 6-month pilot period, DA usage data were collected to assess uptake and completion of the DA. Forty-two patients received the DA, of which 28 patients used the DA, leading to a participation rate of 67%. Patients received the DA after the HPs discussed the LDI and assessed burn depth diagnosis. On average, patients spent 49 minutes on the DA in multiple sessions. Of the patients who logged in, 93% reached the summary page, indicating that they had completed the DA.

A sample of patients who had used the DA was interviewed. Overall, patients were very positive about the DA and found it helpful. However, 1 patient found the DA redundant as the patient already had a clear view regarding the preferred treatment (Table 4). Patients indicated that having additional information was useful and that the option of involving relatives was valuable. However, a patient mentioned feeling overwhelmed by everything that happened after sustaining burns, meaning that not all the information was fully absorbed. The interviewed patients indicated that they felt they had made the treatment decision together with the burn physician.

In the evaluating focus groups, HPs were positive about the use of the DA and mentioned several advantages, including increased patient motivation and autonomy, and time-saving when explaining different treatment options. They acknowledged that it is beneficial for patients to have clear information on the possible treatment options, as this helps them to better understand the decision made. However, they also mentioned several barriers, including the fact that many patients lack the literacy skills required to use the DA, and that logistical issues sometimes prevent HPs from using the DA to its full potential. HPs from all 3 burn centers mentioned that they felt SDM was at the same level as before implementation or had improved (Table 4).

Finally, possible improvements to the DA were discussed with the HPs, who mentioned that outcome information would improve the DA and would support patients in decision-making. Outcome information on scar satisfaction, scar elasticity, number of dressing changes, and donor site satisfaction were deemed interesting.

**Table 4.** Quotes from patients and health care professionals on the use of the decision aid.

Theme	Respondent	Quote
Relevance of the decision aid	Patient	“It looks very nice, but I already had my opinion ready, so in principle it was a bit redundant.”
Involving relatives	Patient	“It was rapidly made clear that I could make a treatment decision and in this decision my wife was also included, which was very pleasant.”
Shared decision-making	Patient	“I made the decision together with my burn physician. It was not the case that the burn physician gave advice and left it completely to me to make the decision, but they did mention very clearly the advantages and disadvantages of the treatment and they also became evident when using the DA.”
Shared decision-making	Health care professional	“The DA improved SDM with the patient. Patients who are capable of using the DA are more equal conversation partners and are able to make a well-informed decision based on the information provided.”
Shared decision-making	Health care professional	“If you can take the time to use the DA and hand it to the patients at the right time, then it most definitely improves SDM.”

#### Level of Normalization of the DA

**Figure 2.**
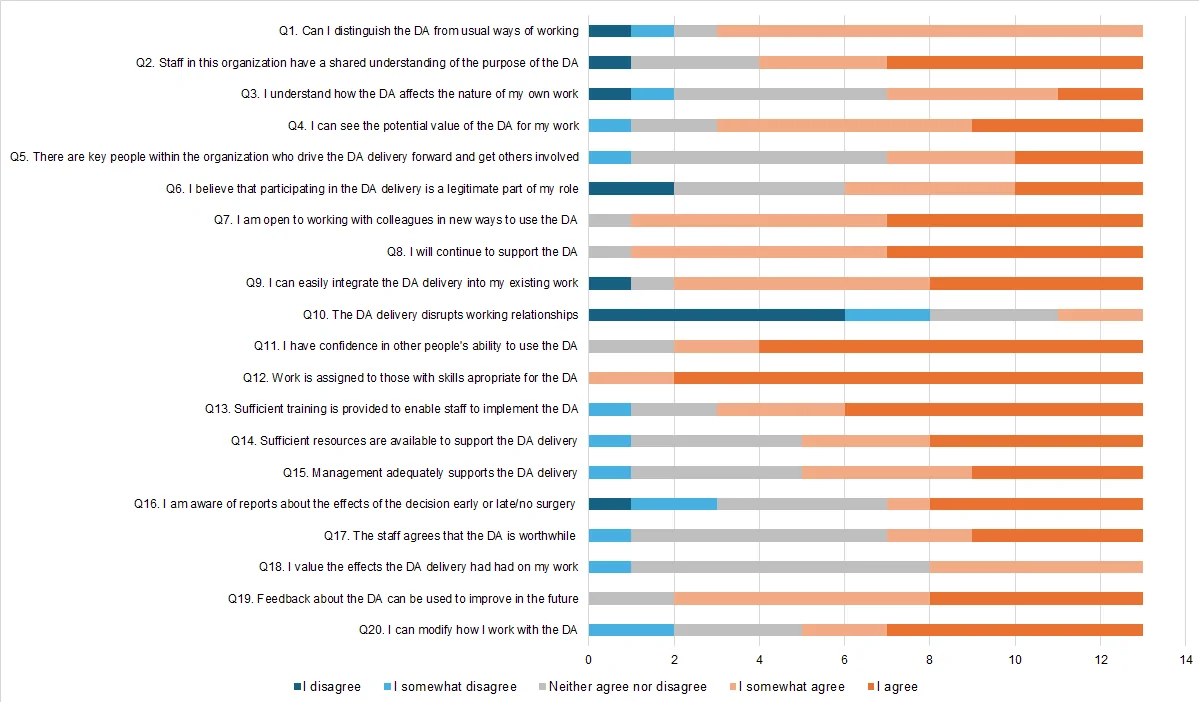
Normalization Measure Development questionnaire (NOMAD) to determine the level of normalization of the DA after implementation among health care professionals. DA: decision aid.

To evaluate HPs’ perceptions of the implementation, adaptation, and impact of the DA in daily practice, the NOMAD questionnaire was distributed to the implementation teams (n=26), 13 of whom participated (50% response rate). Of the 13 HPs, 61.5% (8/13) were burn physicians, 15.4% (2/13) nurse specialists, 15.4% (2/13) nurses, and 7.7% (1/13) residents. The NOMAD survey revealed that HPs agreed that the DA had led to a change in work processes (10/13, 76.9%, agreed with question 1 and 9/13, 69.2%, with question 3), and the respondents saw the added value of the DA in comparison to usual care (10/13, 76.9%, agreed with question 4; Figure 2). HPs also indicated that they now consider the use of the DA as part of their responsibilities (7/13, 53.8%, agreed with question 6) and are open to support and keep using the DA in their daily work (12/13, 92.3%, agreed with questions 7 and 8). HPs consider the integration of the DA into their work process as easy (11/13, 84.7%, agreed with question 9) and have a positive attitude toward the skills and use of their colleagues (84.6% agreed with question 11 and 13/13, 100%, agreed with question 12). Training and resources were deemed sufficient by 76.9% (10/13) of respondents (questions 13 and 14). However, the key stakeholders within the organization were not fully clear to the HPs (7/13, 53.9%, disagreed or were unsure with question 5). Finally, it is believed that using the DA will improve the decision-making process (11/13, 84.7%, agreed with question 19).

## Discussion

### Principal Findings

In this study, we developed and implemented the first DA to support SDM between patients and HPs regarding the treatment of deep partial-thickness burns. The DA supports decisions about early vs “late or no” surgery and includes a paper handout sheet, an interactive website, and a summary sheet capturing patients’ values and preferences. The DA provides a uniform and clear source of information and supports SDM between patients and HPs. The DA has been implemented, and there is a positive attitude toward its future use.

Previous studies have shown that most patients wish to be involved in clinical decision-making, although the desired level of involvement varies substantially between individuals [17,31]. This aligns with our needs assessment, in which some patients expressed a preference for active participation while others preferred to rely on the HP’s recommendation. These differences underline that SDM in burn care must be inherently patient-centered, with information and support tailored to individual preferences and capacities rather than assuming a uniform level of involvement. A DA increases knowledge of the different treatment options and can facilitate information provision [17]. By offering layered information via the paper handout sheet, the interactive website, and a summary sheet, the DA allows patients to access and revisit content at their own pace and to select sections that are most relevant to their personal situation and informational needs.

Health literacy is increasingly recognized as a critical determinant of whether patients are able to participate effectively in SDM [32]. A recent analysis of 198 DAs showed that although most DAs perform well in terms of layout and visual presentation, many fall short in providing understandable content, such as defining medical terminology or using a short and simple sentence structure [33]. Moreover, only a minority of these DAs were cocreated with patients with low health literacy. Similarly, in our research, the patients participating in the co-design sessions did not have low health literacy, which may have limited the extent to which the DA reflects the needs of this group. We improved accessibility by writing the text in common Dutch (B1 level), using short sentences and bullet points, and adding illustrations to clarify the provided information. In burn care, challenges extend beyond health literacy alone, as many patients have a language barrier and the DA contains an online component which may pose barriers for patients with limited digital skills [34]. We intended to improve accessibility for patients with low health literacy and digital literacy by stimulating support from relatives or HPs in clinic. However, future refinement of the DA should explore additional formats, such as video animations, multilingual versions, or more extensive clinical support, to better accommodate patients with lower health and digital literacy [35,36]. Despite these challenges, uptake of the DA was relatively high, and most patients who started the DA also completed it.

The implementation of a DA might face different barriers and facilitators, as was demonstrated by the systematic review of Waddell et al [12]. The identified barriers were mostly logistic-related, such as the perception that acuity limits SDM, or a lack of space and time. This matched the identified barrier on infrastructure in our acceptability test. Especially facilitating adequate time for a patient to use the DA was difficult due to the logistics of acute patient care. On the other hand, HPs acknowledged that the DA could ultimately save time by structuring explanations about the different treatment options.

The NOMAD questionnaire showed that, despite several anticipated barriers before implementation, HPs perceived clear added value of the DA compared to usual care. The relatively high normalization potential suggests that HPs saw clear added value and felt the DA could be integrated into daily clinical practice. This suggests that the DA was successfully integrated into the existing workflow, which likely reflects the active involvement of the implementation team. HPs reported that incorporating the DA into their work processes was straightforward, which aligns with the efforts made to optimize logistics in the implementation strategy, such as mapping the patient journey, dividing tasks and responsibilities, and having interim evaluation meetings. These strategies align with implementation literature emphasizing structured process mapping, team coordination, and early involvement of stakeholders as facilitators of workflow integration for DAs in daily clinical practice [37].

Visible leadership has been identified as an important factor for sustained use of SDM and DAs [38]. In our study, HPs reported that the role of key stakeholders was not fully clear and that stronger institutional involvement would likely further support sustained use of the DA. Future efforts should address factors such as appointing local stakeholders or early adopters, embedding the DA in formal protocols, and ensuring that management recognizes and supports the time and resources needed for the use of the DA. Particularly in acute burn care, this is important as high workload and operational schedules can easily overrule SDM.

A recent systematic review and meta-analysis suggests that patient DAs developed using systematic, user-centered processes, which explicitly involve patients, tend to be of a higher quality and are associated with better patient outcomes [39]. Collaboration between the steering and implementation groups, HPs, and patient panels ensured that all relevant perspectives were integrated into the DA. This integration likely contributed to the DA’s perceived relevance and usability for both patients and HPs. At the same time, the relatively small number of patient participants in the co-design sessions might introduce a power imbalance. Participation required substantial time investment and preparatory work, which limited the number of patients able to participate. However, the participating patient representatives were highly experienced, and the co-design session leaders ensured equal contribution between patients and HPs.

To enhance the DA, subsequent research is desired to add outcome information [40]. Therefore, we are currently undertaking a multicenter prospective study to gain information regarding scar quality, clinical outcomes, and patient-reported outcomes for both early surgery and “late or no” surgery in deep partial-thickness burns [4]. This will provide up-to-date and evidence-based information on outcomes, helping patients to better understand possible risks and compare treatment expectations [41]. Incorporating outcomes, such as scar elasticity, satisfaction with scar and donor site, number of dressing changes and surgeries, and broader patient-reported outcomes such as quality of life and return to work, can enhance the DA and can be meaningful for the treatment decision. The correct visual representation of the outcome data should also be discussed, as it allows patients to fully understand the information provided.

Several limitations of this study should be considered. The number of patients participating in phase 2 co-design sessions and in the pilot use of the DA was modest, partly due to the time required for participation and lower than expected admissions during the pilot period, which may limit representativeness of patient experiences. In addition, this study did not include a formal effectiveness evaluation of the DA’s impact on SDM, so conclusions about effectiveness rely on qualitative data from interviews with patients and focus groups with HPs, rather than controlled comparisons.

Finally, the DA was developed specifically for patients with deep partial-thickness burns, in whom the treatment decision is clinically relevant. It does not explicitly incorporate other burn characteristics, such as different burn depth or specific burn size. As burn and patient characteristics can significantly influence course, treatment, and outcome, clinicians should use the DA in combination with clinical judgment to determine its applicability to individual patients, particularly in cases involving extensive burns or other relevant clinical scenarios.

### Conclusions

This study outlines a comprehensive, user-centered approach for developing and implementing a DA in acute burn care, integrating patients and HPs throughout the whole process. Our findings suggest that, when clinical teams are involved early and implementation strategies such as patient-journey mapping, role clarification, and iterative feedback are used, a DA can be normalized within the high-pressure context of acute burn care and support more patient-centered treatment decisions. The DA enhances access to uniform information, helps clarify patient values and preferences, and supports perceived SDM, while also highlighting ongoing challenges around health literacy, digital access, and organizational support. Its successful integration offers a model for implementing a DA in other acute clinical contexts with time pressure and complex decisions.

## Supplementary material

10.2196/86789 Multimedia Appendix 1 Needs assessment survey for health care providers.

10.2196/86789 Multimedia Appendix 2 Analysis of open-ended survey questions among health care professionals.

10.2196/86789 Checklist 1 COREQ Checklist 1

## Abbreviations

CFIR: Consolidated Framework for Implementation Research
COREQ: Consolidated Criteria for Reporting Qualitative Research
DA: decision aid
HP: health care professional
LDI: laser Doppler imaging
MEC-U: Medical Research Ethics Committees United
NOMAD: Normalization Measure Development
SDM: shared decision-making
