# Supplementary material for: Shared Decision-Making in the Management of Deep Partial-Thickness Burns: Development and Implementation of a Multicomponent Decision Aid
Source: J Med Internet Res. 2026 Sep 4;28:e86789. doi: 10.2196/86789 (PMC13544618; doi:10.2196/86789)
Supplement: Supplementary file 2 [file jmir-v28-e86789-s001.docx]

**The choice of treatment for burns**

Dear healthcare provider,

By filling out this questionnaire you contribute to the development of a decision aid in order to support shared decision-making regarding the treatment choice of having surgery or not for deep partial-thickness burns.

We aim to develop a nationally supported decision aid that contributes to shared decision-making, which makes:

- The practitioners better understand the person behind the patient;
- Patients more aware of having a choice and their role in this choice;
- Patients better informed about the treatment options and potential risks;
- Patients able to indicate what is important to them;
- That patients can make a treatment decision together with their practitioner.

We do this together with the experts of ZorgKeuzelab who have extensive experience in digital decision aids (www.zorgkeuzelab.nl). Your answers will be included in the development of the decision aid. The question are about your experiences with information provision, the decision process, and shared decision-making. About your preferences for a decision aid and how this can be improved.

Completing the survey takes 15 to 20 minutes. We appreciate your contribution!

**Consent**

I hereby declare that I am well informed about the questionnaire and its purpose, as explained above. Any questions were answered satisfactorily and I had enough time to decide whether or not to participate.

I know that participating is voluntary and I also know that at any moment I can decide not to participate. I do not have to give a reason. I know that the questions that I have already completed until I decided to stop can be used for the study. I know that my answered will be used coded.

I give permission to keep my answers at the research site for 15 years.

- I agree to participate in this study.

**About you**

1. What is your position?

- Burn physician
- Trauma surgeon
- Plastic surgeon
- Resident
- Nurse
- Nurse specialist
- Team leader
- Medical manager
- Other:

2. For how many years have you been working in this specialty?

- 5 years or less
- More than 5 years

3. In which hospital are you employed?

- Red Cross Hospital Beverwijk
- Maasstad Hospital Rotterdam
- Martini Hospital Groningen

**About your work location**

1. On average, how many adult patients with burns do you see per week?

- Not applicable
- 1-5
- 6-10
- 11-15
- 16-20
- > 20

2. With how many of these patients with deep partial-thickness burns do you discuss the treatment option ‘surgical intervention’ and specifically the timing of this (early or late/no surgery)?

- Not applicable
- 1-5
- 6-10
- 11-15
- 16-20
- > 20

3. What are the criteria on which you determine to discuss the option of ‘early or late surgery for a deep partial-thickness burn’ with a patient and his/her family?

4. In your hospital, which healthcare providers are involved in the choice between early or late/no surgery in patients with deep partial-thickness burns? *Multiple options possible*

- Burn physician
- Trauma surgeon
- Plastic surgeon
- Resident
- Nurse
- Nurse specialist
- Team leader
- Medical manager
- Other:

**Your experiences regarding information provision, the decision process and shared decision-making**

5. To what extend are you satisfied with the current provision of information (e.g., leaflets or websites with information) for yourself about the option of early or late/no surgery in patients with deep partial-thickness burns? *5-point scale, 1: Not at all satisfied – 5: Very satisfied*

○ Not at all satisfied ○ Not satisfied ○ Not dissatisfied nor satisfied ○ Satisfied ○ Very satisfied

6. In your hospital, who informs patients with burns (and their family) about the treatment decision ‘early or late/no surgery in patients with deep partial-thickness burns’? *Multiple options possible*

- Burn physician
- Trauma surgeon
- Plastic surgeon
- Resident
- Nurse
- Nurse specialist
- Team leader
- Medical manager
- Other:

7. In your opinion, what is the main source of information for patients (and their family) about the treatment option ‘early or late/no surgery in patients with deep partial-thickness burns’?

8. To what extend do you agree with the following statements (*1: Not at all satisfied – 5: Very satisfied*):

a. Patients with deep partial-thickness burns (and their families) are adequately informed in my hospital about early or late/no surgery and its timing.
b. The information available to patients (and their families) about early and late/no surgery options is reliable.

9. By whom is the final decision on ‘early or late/no surgery in patients with deep partial-thickness burns’ usually made now? *Multiple options possible*

- The patient
- Family
- Burn physician
- Trauma surgeon
- Plastic surgeon
- Resident
- Nurse
- Nurse specialist
- Other:

**Your perceptions about shared decision-making**

10. To what extent do you agree with the following statements (*1: Not at all satisfied – 5: Very satisfied*):

- Better informed patients (and family) facilitate the decision-making process
- Better informed patients (and family) complicate the decision-making process
- If a patient (and family) does not want to be involved in the decision of ‘early or late/no surgery’, a physician or other healthcare professional should still try to involve the patient

11. The final decision for ‘early or late/no surgery’ should ideally be made by:

- de patient/family
- de patient/family after hearing and considering the physician’s opinion
- the physician and the patient/family together
- the physician after hearing and considering the opinion of the patient/family
- the physician

12. Do you believe that all possible disadvantages/complications of both options ‘early or late/no surgery’ should be mentioned to the patient (even if the probability of their occurrence is small) *(1: never – 5: always)*?

○ Never ○ Sometimes ○ Regularly ○ Often ○ Always

13. What do you experience as barriers/facilitators to involving patients in decision-making?

**Perceptions and desires regarding the online decision aid**

14. Do you already have experiences with referring to decision aids? *Yes / no*

15. If yes, how was your experience?

16. A decision aid for ‘early or late/no surgery’ seems to me:

- 1: Not at all desirable – 5: Very desirable
- Please explain:

17. What do you think would be the biggest advantage of a decision aid for choosing between early or late/no surgery?

18. What do you think would be the biggest disadvantage of a decision aid for choosing between early or late/no surgery?

19. Who do you think is the most appropriate healthcare professional to guide the patient and family in using the decision aid for ‘early or late/no surgery’ in patients with deep partial-thickness burns (including explaining and discussing the summary)? *Multiple options possible*

- Burn physician
- Trauma surgeon
- Plastic surgeon
- Resident
- Nurse
- Nurse specialist
- Other:

20. What are the main questions patients and their families ask you regarding the treatment options ‘early or late/no surgery’?

21. What are the most common misunderstandings among patients and their families regarding the treatment options ‘early or late/no surgery’?

22. What do you think should at least be covered in the decision aid for ‘early or late/no surgery’?

**Closing**

Do you have any additional comments in response to this survey and/or regarding the decision aid?

May we approach you for a possible follow-up survey? *Ja 🡪 personal data / No*
