# Supplementary material for: Shared Decision-Making in the Management of Deep Partial-Thickness Burns: Development and Implementation of a Multicomponent Decision Aid
Source: J Med Internet Res. 2026 Sep 4;28:e86789. doi: 10.2196/86789 (PMC13544618; doi:10.2196/86789)
Supplement: Supplementary file 3 [file jmir-v28-e86789-s002.docx]

Analysis of open-ended survey questions among healthcare professionals

|  |  | ***n*** |
| --- | --- | --- |
|  | **What are the criteria on which you determine to discuss treatment options for deep partial-thickness burns with a patient and his/her family?*** | **11** |
|  | Severity of the burn | 10 |
|  | Preferences of the patient | 4 |
|  | Overall health status of the patient | 4 |
|  | Work or hobbies | 2 |
|  | Pain | 2 |
|  | **What is the main source of information for patients about the treatment options for deep partial-thickness burns?*** | **35** |
|  | Healthcare professional | 34 |
|  | Information leaflet | 3 |
|  | Digital information | 2 |
|  | Information is not provided | 1 |
|  | Literature | 1 |
|  | **What are possible barriers or facilitators when involving patients in decision-making?** | **30** |
|  | Patient is not able to use the decision aid due to medical condition | 8 |
|  | Patient does not fully understand the information provided | 7 |
|  | HP is not able to perform shared decision-making | 6 |
|  | Patient receives to much information | 6 |
|  | Patient receives unclear information | 6 |
|  | Language barrier | 5 |
|  | Decision of patient and HP do not align | 3 |
|  | Patient does not want to make a shared decision | 2 |
|  | Patient has limited insight into the disease process | 2 |
|  | Patient does not have enough time to use the DA | 1 |
|  | **What was your experience referring to decision aids?*** | **6** |
|  | Positive experience | 5 |
|  | Technically complicated | 2 |
|  | Not suitable for every patient | 1 |
|  | **What is the biggest advantage of using a decision aid for treatment decisions in deep partial-thickness burns?*** | **32** |
|  | Helps the patient to make a decision | 21 |
|  | Provides the right information for the patient | 13 |
|  | More autonomy for the patient | 7 |
|  | Results in a better tailored treatment | 2 |
|  | **What is the biggest disadvantage of a decision aid for treatment decisions in deep partial-thickness burns?*** | **27** |
| Patient receives too much or unclear information | | 10 |
|  | Places to much responsibility on patients | 8 |
|  | Not suitable for every patients | 6 |
|  | Logistic inconveniences | 4 |
|  | Decision of patient and HP do not align | 2 |
|  |  |  |
|  | Patient does not have enough time to use the DA | 2 |
|  | **What are the main questions patients and their family ask you regarding the treatment of deep partial-thickness burns?*** | **23** |
|  | Questions regarding time to wound healing | 13 |
|  | If a patient will return to their previous level of functioning | 8 |
|  | Questions regarding scarring | 6 |
|  | Questions regarding complications | 6 |
|  | What the choice of the HP would be | 5 |
|  | General questions on advantages and disadvantages of the treatment options | 4 |
|  | **What are the most common misunderstandings among patients and their family regarding the treatment of deep partial-thickness burns?*** | **13** |
|  | Procedure of surgery is misunderstood | 4 |
|  | Scar quality | 4 |
|  | Patient believes there is no treatment decision to make | 2 |
|  | Healing without treatment is best | 1 |
|  | Patient is solely responsible to make a treatment decision | 1 |
|  | Believing a patient can directly go home after surgery | 1 |
|  | No logistic possibility for early surgery | 1 |
|  | **What should at least be covered in the decision aid regarding the treatment of deep partial-thickness burns?*** | **28** |
|  | General advantages and disadvantages of the treatment options | 16 |
|  | Scar quality | 10 |
|  | Expected recovery time for burn wound and donor site | 8 |
|  | Complications and risks of treatment | 6 |
|  | Number of surgeries per treatment options | 5 |
|  | Length of hospital stay | 3 |
|  | Possibility of returning to previous level of functioning | 3 |
|  | What is most important in the patient’s life | 2 |
|  | Scientific literature | 2 |
|  | Number of wound dressing changes | 2 |
|  | The choice can also be made by the HP | 1 |
|  | Alternative treatments | 1 |

**Multiple answers possible*
